# Ultra-Fast Sub-Minute Brain MRI with Deep-Learning-reconstruction for Anesthesia-Free Emergency Imaging in Children

**DOI:** 10.1007/s00062-026-01627-7

**Published:** 2026-02-19

**Authors:** Sebastian Altmann, Nils F Grauhan, Mario A A Mercado, Haidara Almansour, Roman H Paul, Vannessa Ines Schöffling, Malte Ottenhausen, Marc A Brockmann, Ahmed E Othman

**Affiliations:** 1 Department of Neuroradiology, University Medical Center of the Johannes Gutenberg University Mainz, Mainz, Germany; 2 Institute of Medical Biostatistics, Epidemiology and Informatics, University Medical Center of the Johannes Gutenberg University Mainz, Mainz, Germany; 3 Department of Neuroradiology, University Medical Center of the Johannes Gutenberg University Mainz, Mainz, Germany; 4 Department of Pediatric Neurosurgery, University Medical Center of the Johannes Gutenberg University Mainz, Mainz, Germany

**Keywords:** Ultra-fast pediatric brain MRI, Deep-Learning reconstruction, MRI without anaesthesia, Pediatric emergencies

## Abstract

**Purpose:**

As MRI frequently requires general anesthesia in pediatric patients, there is an undersupply in clinical routine. This may result in delayed examinations or use of CT, especially in emergency settings. To enable ad-hoc MRI scans and rule out increased intracranial pressure or intracranial mass lesions, we combined various acceleration techniques with Deep-Learning reconstruction, generating ultra-fast diagnostic images sufficient to exclude critical pathologies.

**Methods:**

Thirty-six MRI datasets of infants with a median age of 35.2 months (SD ± 23.2) and anesthesia-free imaging were retrospectively evaluated. Imaging was performed using ultra-fast T2-weighted sequences in three planes (slice thickness 5 mm; total acquisition time 47s). Four readers evaluated subjective image quality using a 5-point Likert-scale. Readers were asked to indicate how safe they felt about assessing possible midline displacement or mass lesion. For semi-quantitative analysis, readers reported diameters of lateral and third ventricles. Gwet’s AC2 and intraclass correlation (ICC) were used for interrater agreement.

**Results:**

94.4% of datasets showed at least acceptable diagnostic confidence. Readers felt confident excluding acute intracranial pathology. 52.1% of ultra-fast sequences demonstrated good to excellent image quality. 72.6% were rated with good or excellent diagnostic confidence. Interrater reliability demonstrated almost excellent agreement of diagnostic confidence (Gwet’s AC2 ≥ 0.886) and image quality (Gwet’s AC2 ≥ 0.942). Excellent agreement regarding ventricular width (ICC values ≥ 0.966) was shown for all measurements.

**Conclusion:**

The use of deep-learning reconstruction algorithms in pediatric brain MRI is feasible, allowing anesthesia-free emergency imaging. This will reduce periprocedural risk, number of necessary CT scans, and lower healthcare costs.

**Supplementary Information:**

The online version of this article (10.1007/s00062-026-01627-7) contains supplementary material, which is available to authorized users.

## Introduction

MRI in infants often demands general anesthesia, resulting in higher healthcare costs and undersupply of pediatric brain MRIs in clinical routine. For this reason, various efforts have been made to speed up brain MRI protocols over the years. Nevertheless, until recently, conventional techniques have had limitations. Although established methods compensate for small periodic movements, they require longer scan times, limiting their use in pediatric subgroups. However, they cannot compensate for excessive motion artefacts caused by screaming or excessive agitation. Although markedly reduced acquisition times of T2-weighted datasets have been achieved, e.g. with real-time MRI, generating diagnostic images without anesthesia remains challenging. These techniques often suffer from reduced image quality with minor resolution due to minor matrix size or limited T2 contrast [1–4]. First feasibility studies could also demonstrate that acquiring a full multi-contrast dataset on 3T MRI is feasible in about one and a half minutes [5]. Moreover, implementing new approaches to motion correction enables robust artefact reduction. Especially by combining new techniques in multi-layered MRI protocols, it is increasingly possible to reduce the need for anesthesia in pediatric MRI [6].

Furthermore, license purchases and necessary hardware are quite expensive. Thus, this technique is only applicable to some scanners. Besides the shortening of acquisition times, supplementary techniques can be used to prevent or minimize movement artefacts during anesthesia-free examinations. For example, the feed and wrap technique is essential in pediatric radiology to keep children calm during scans and minimize movement artefacts. Still, its efficacy is most often limited [7]. Furthermore, image acquisition can be challenging in children with cognitive impairment, even into adulthood. This can be improved by playfully introducing the children to the clinical settings of MRI [8, 9]. However, this cannot be maintained in emergency workups where immediate examinations are necessary and thus, MRI with anesthesia or even CT is used.

Using Deep-learning-accelerated brain MRI combined with various acceleration methods, our study introduces a novel approach to speed up image acquisition as much as possible while maintaining diagnostic quality. We hypothesize that ultra-fast brain MRI with Deep-Learning reconstruction allows image acquisition within seconds and is feasible in clinical routine, enabling emergency MRI examinations of infants and children without anesthesia.

## Material and Methods

### Ethics Approval Declaration

This study was conducted in accordance with the Declaration of Helsinki and approved by the Ethics Committee of the Rhineland-Palatinate Chamber of Physicians (approval no. 2024–17542). Written informed consent was waived by the Institutional Review Board. No funding was received.

### Study Sample

Between June 2023 and October 2024, 38 consecutive children with a clinical indication for brain MRI to exclude an (i) acute intracranial pathology or (ii) CSF circulatory dysfunction were subjected to a contrast-free brain MRI with ultra-fast sequences and were retrospectively evaluated. Inclusion criteria were i) clinical indication for brain MRI, ii) patient age between 0 and 6 years ii) fully acquired dataset of ultra-fast T2-weighted datasets in all three planes. Two patients were excluded due to severe agitation and could not be imaged, resulting in the need for further follow-up. Furthermore, one patient was excluded due to excessive artifacts through a cochlear implant. The final study sample resulted in 36 datasets from 35 patients (one patient imaged twice within the course of the clinical follow up).

### Image Acquisition

Imaging was performed with a 1.5‑T clinical scanner (Magnetom Sola; Siemens Healthcare) using a 20-channel head-neck coil. Patients were examined without anesthesia and underwent deep-learning accelerated novel ultra-fast T2 weighted sequences based on the CE-certified “Deep-Resolve-Boost-Techniques” as described in previously published work [10–12]. Sequences were acquired in all three planes (axial, sagittal, coronal), with a slice thickness of 5 mm (cumulative time of acquisition was 47 s for all three planes), and care was taken to ensure that every section of the neurocranium was recorded in at least one plane. Sequence parameters are given in Table 1. Thereby, the applied ultra-fast protocol was 4.3 times faster than a comparable conventional dataset with similar resolution and slice thickness, which would have required 202 s. If the child tolerated the examination well, additional sequences could be included depending on the clinical question. However, these additional sequences were not given to the readers and were not considered in the study analysis.

**Table 1:** Sequence parameters of the ultrafast deep-learning-based protocol; Interpolation (i), Time to Echo (TE), Repetition Time (TR), Simultaneous Multi-Slice (SMS)

Weighting	T2	T2	T2
Orientation	Transversal	Coronal	Sagittal
Averages	1	1	1
Total Time (sec)	17	15	15
Time of Acquisition	5	5	5
FOV (mm^2^)	240	240	240
Matrix size	384 × 384	384 × 384	384 × 384
Phase resolution	80%	80%	80%
TE (ms)	97	97	97
TR (ms)	3850	3470	3470
Reconstructed voxel size (mm^3^)	0.3 × 0.3 × 5 (i)	0.3 × 0.3 × 5 (i)	0.3 × 0.3 × 5 (i)
Parallel imaging acceleration	4	4	4
SMS factor	2	2	2
Slice thickness (mm)	5	5	5
Slice distance factor	10%	10%	10%

### Image Evaluation

Four readers (HA) with seven-years experience in radiology, NFG with eight-years experience in radiology, SA and MAAM with nine-years experience in radiology), independently evaluated subjective image quality. A 5-point Likert-scale was used to assess overall image quality, and diagnostic confidence, regarding the presence of midline displacement, space-occupying lesion or hemorrhage and the evaluation of the ventricular width. Furthermore the occurence of image artifacts was evaluated (Table 2). For semi-quantitative analysis, readers had to assess ventricular width by reporting the diameter of both the lateral and the third ventricle.

**Table 2:** Subjective image analysis

*Grading*	Overall Image quality	
*1*	Very poor image quality	Non-diagnostic, re-scanning necessary
*2*	Poor image quality	Limited anatomical delineation, diagnostic limitations
*3*	Accaptable image quality	Anatomical structures, including the ventricle, brainstem, foramen magnum, and cortex, are distinguishable and can be assessed for intracranial space-occupying lesions and increased intracranial pressure; image quality in this respect poses no relevant diagnostic limitations
*4*	Good image quality	Anatomical structures, including the ventricle, brainstem, foramen magnum, and cortex, are distinguishable and can be assessed for intracranial space-occupying lesions and increased intracranial pressure; image quality in this respect poses no diagnostic limitations
*5*	Excellent image quality	Anatomical structures, including the ventricle, brainstem, foramen magnum, and cortex, are clearly distinguishable and can be assessed for intracranial space-occupying lesions and increased intracranial pressure; image quality in this respect poses no diagnostic limitations. Image quality enables the detection of even smaller intracranial structures

### Statistical Analysis

Statistical analysis was performed using SAS 9.4 (SAS Institute, Cary NC). Continuous variables were reported with mean and standard deviation, and ordinal variables were reported using the median and the interquartile range. Gwet’s AC2 and two-way random single score intraclass correlation (ICC) (2.1), according to Shrout and Fleiss [13] were performed to assess interrater agreement. According to Landis and Koch, Gwet’s AC values of 0.00 to 0.20, 0.21 to 0.40, 0.41 to 0.60, 0.61 to 0.80, and 0.81 to 100 indicate poor, slight, fair, moderate, substantial, and almost perfect agreement levels respectively [14]. The intraclass correlation coefficient was defined as follows: poor, < 0.5; moderate, 0.5–0.75; good, 0.76–0.9; excellent, > 0.9 [15]. For correlation analysis spearman’s roh was applied. *P*-values of less than 0.05 were considered statistically significant.

## Results

### Patient Data

**Fig. 1:**
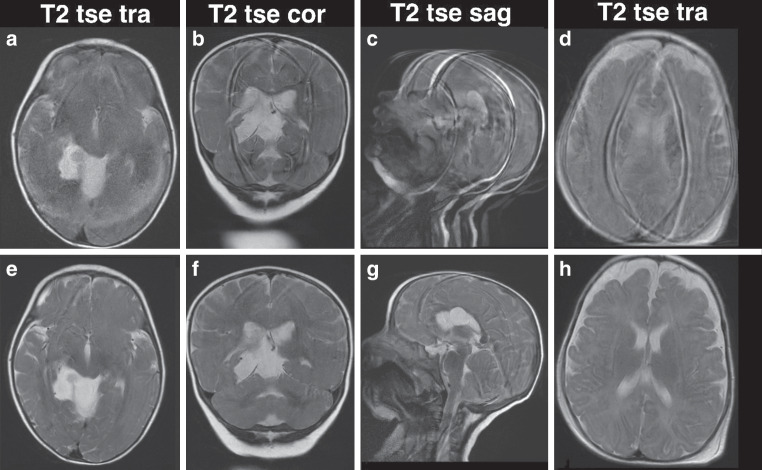
Selective images of patients who required partial re-scanning due to apparent movement artefacts. An11-month-old girl after fenestration of a large arachnoid cyst (**a**–**d**), a 12-month-old boy with an incidental finding of the intraventricular cyst (**e**–**f**), a 4-month old baby, who was dropped (**g**–**h**). Turbo-spin-echo (tse), transversal (tra), coronal (cor), sagittal (sag)

Ultra-fast sequences were successfully acquired in 36 cases with a mean age of 35.2 months (SD ± 23.2). In 13 patients, single sequences had to be repeated due to initial motion artefacts. Figure 1 presents examinations that were immediately repeated due to initial motion artefacts. The clinical indications were: routine examination for evaluation of ventricular width (*n* = 16), traumatic brain injury (*n* = 10), suspicion of increased intracranial pressure (*n* = 6), seizure (*n* = 2), reduced vigilance (*n* = 1), paresis of the right arm (*n* = 1). The findings were: normal brain MRI or known congenital abnormalities (*n* = 25), intracranial mass lesion (*n* = 7), signs of overdrainage due to shunt dysfunction (1), extracranial hematoma (*n* = 1), postischemic defect (*n* = 1), hydrocephalus (*n* = 1).

### Subjective and Semiquantitative Image Comparison

**Fig. 2:**
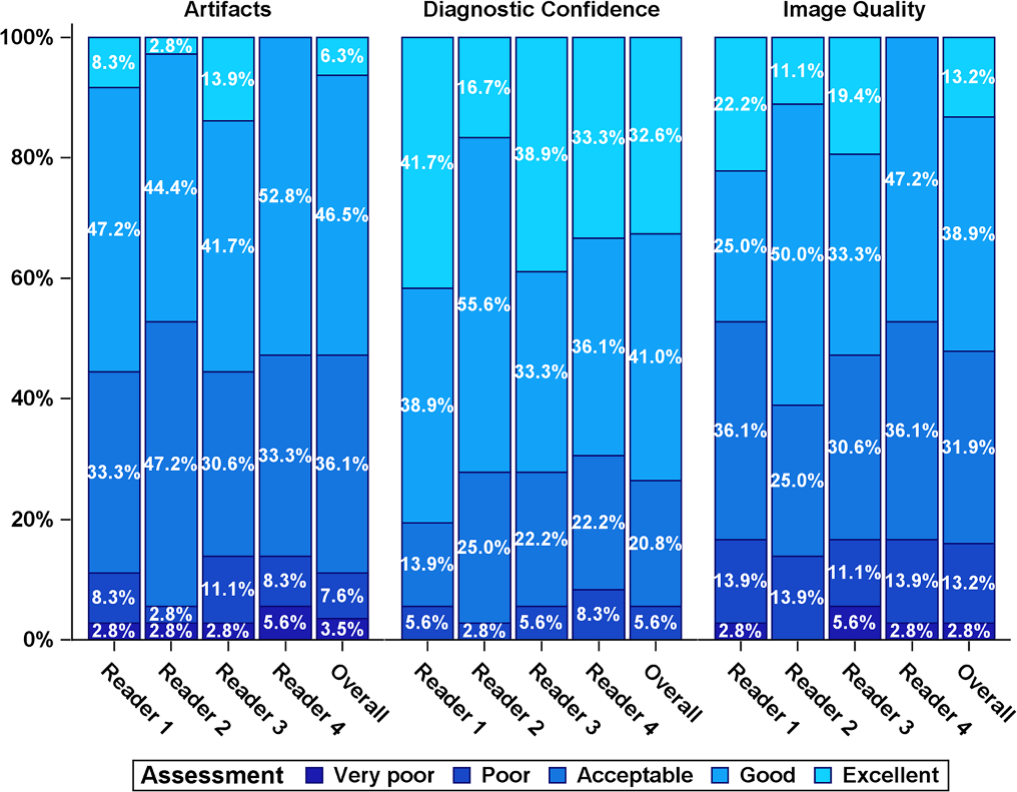
Bar graphs showing the results of the analysis of artifacts, diagnostic confidence and image quality

**Fig. 3:**
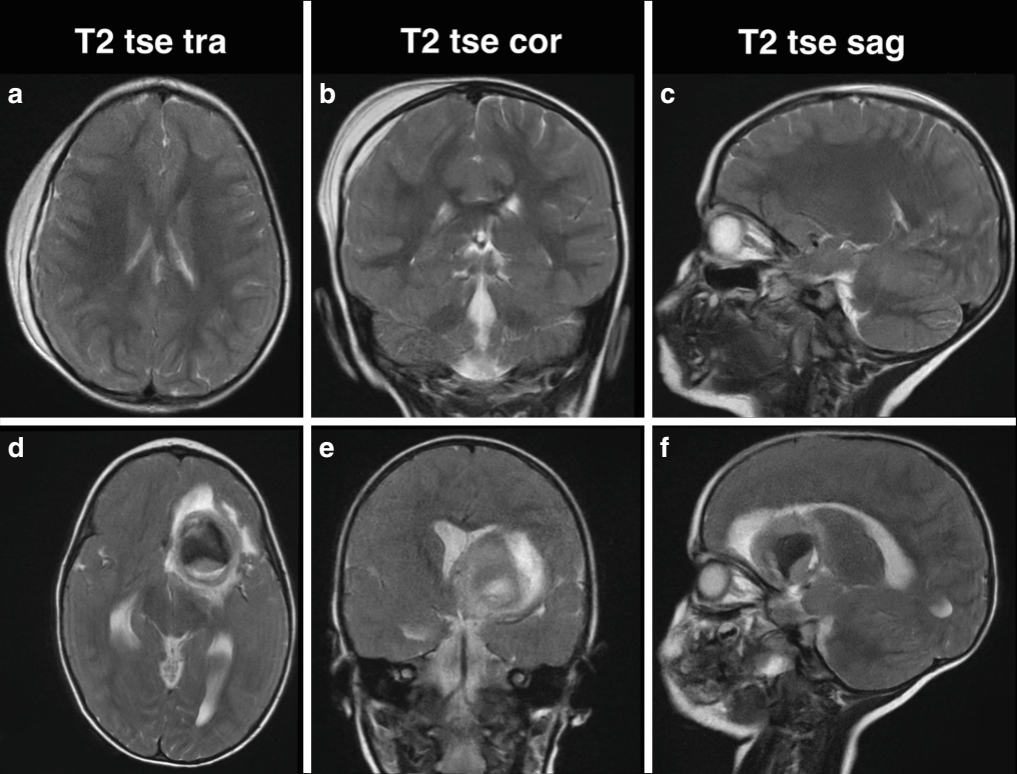
A 30-month-old girl with subdural hematoma who fell off the loft bed (**a**–**c**) and a 21-month-old female with signs of concussion after head trauma and intraparenchymal hemorrhage (**d**–**f**). Turbo-spin-echo (tse), transversal (tra), coronal (cor), sagittal (sag)

**Fig. 4:**
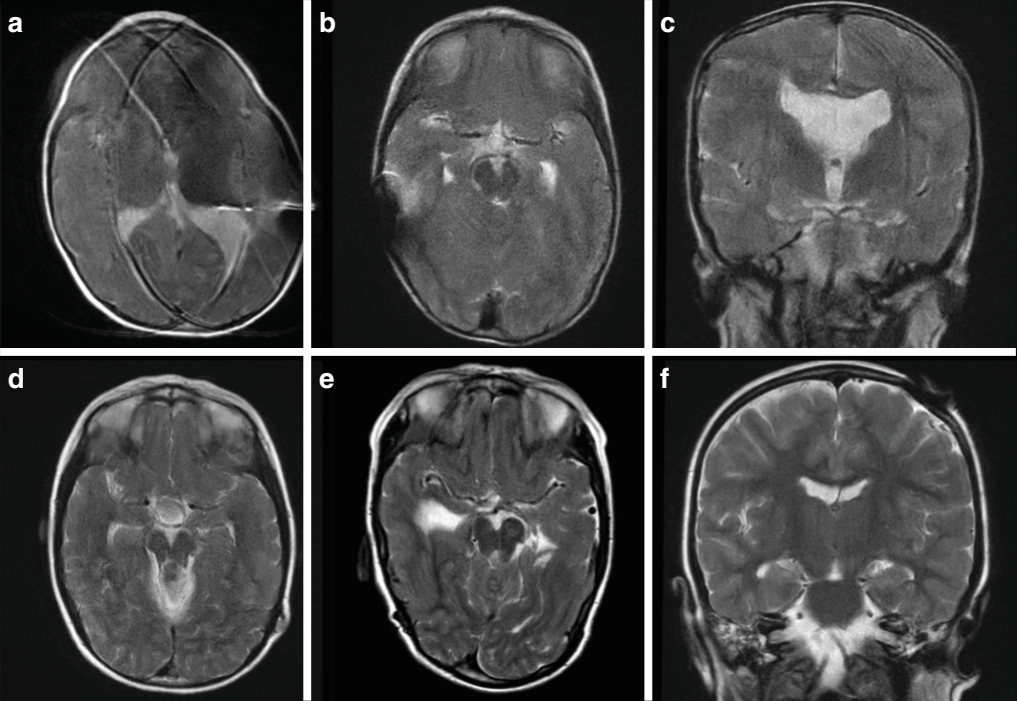
Exemplary images from one reader across different grades of the applied Likert-scale. Transversal T2-weighted image, rated as image quality grade 1 and diagnostic confidence grade 2; transversal T2-weighted image, rated as image quality grade 2 and diagnostic confidence grade 3, coronal T2-weighted image, rated as image quality grade 3 and diagnostic confidence grade 3, transversal T2-weighted image, rated as image quality grade 3 and diagnostic confidence grade 4, transversal T2-weighted image, rated as image quality grade 4 and diagnostic confidence grade 5, coronal T2-weighted image, rated as image quality grade 5 and diagnostic confidence grade 5

Overall 94.4% of datasets showed at least acceptable diagnostic confidence. Two cases demonstrated persistent motion artefacts even after repeating blurred sequences, resulting in difficulties in image interpretation. Nevertheless, examinations did not need to be rescheduled because diagnostic confidence was sufficient to exclude acute intracranial pathology (hemorrhage/signs of increased intracranial pressure). In 52.1% of cases, the ultra-fast sequences demonstrated good to excellent image quality, and 72.6% were rated with good or excellent diagnostic confidence (Fig. 2; Table 3). Figure 3 shows examples of a 2.5-year-old girl with a subdural hematoma and a 21-month-old female with signs of concussion after head trauma and intraparenchymal hemorrhage. Figure 4 presents image examples from one reader across different grades of the applied Likert-scale.

**Table 3:** Subjective and Semiquantitative Image Analysis

Parameter	Reader		
Artifacts	Reader 1	Median (IQR)	3.5 (0.9)
	Reader 2	Median (IQR)	3.4 (0.7)
	Reader 3	Median (IQR)	3.5 (1.0)
	Reader 4	Median (IQR)	3.3 (0.9)
	Overall	Median (IQR)	3.4 (0.9)
Image quality	Reader 1	Median (IQR)	3.0 (1.0)
	Reader 2	Median (IQR)	4.0 (1.0)
	Reader 3	Median (IQR)	4.0 (1.0)
	Reader 4	Median (IQR)	3.0 (1.0)
	Overall	Median (IQR)	4.0 (1.0)
Diagnostic confidence	Reader 1	Median (IQR)	4.0 (1.0)
	Reader 2	Median (IQR)	4.0 (1.0)
	Reader 3	Median (IQR)	4.0 (2.0)
	Reader 4	Median (IQR)	4.0 (2.0)
	Overall	Median (IQR)	4.0 (2.0)
Third ventricle [mm]	Reader 1	Mean (SD)	5.1 (4.0)
	Reader 2	Mean (SD)	5.1 (3.6)
	Reader 3	Mean (SD)	5.1 (4.0)
	Reader 4	Mean (SD)	5.0 (3.9)
	Overall	Mean (SD)	5.1 (3.8)
Left ventricle [mm]	Reader 1	Mean (SD)	8.3 (7.5)
	Reader 2	Mean (SD)	8.4 (7.9)
	Reader 3	Mean (SD)	8.1 (7.3)
	Reader 4	Mean (SD)	7.7 (7.6)
	Overall	Mean (SD)	8.1 (7.5)
Right ventricle [mm]	Reader 1	Mean (SD)	7.4 (8.8)
	Reader 2	Mean (SD)	7.3 (8.1)
	Reader 3	Mean (SD)	7.1 (8.3)
	Reader 4	Mean (SD)	7.2 (8.4)
	Overall	Mean (SD)	7.3 (8.3)

*SD* Standard deviation, *IQR* Interquartile range

**Fig. 5:**
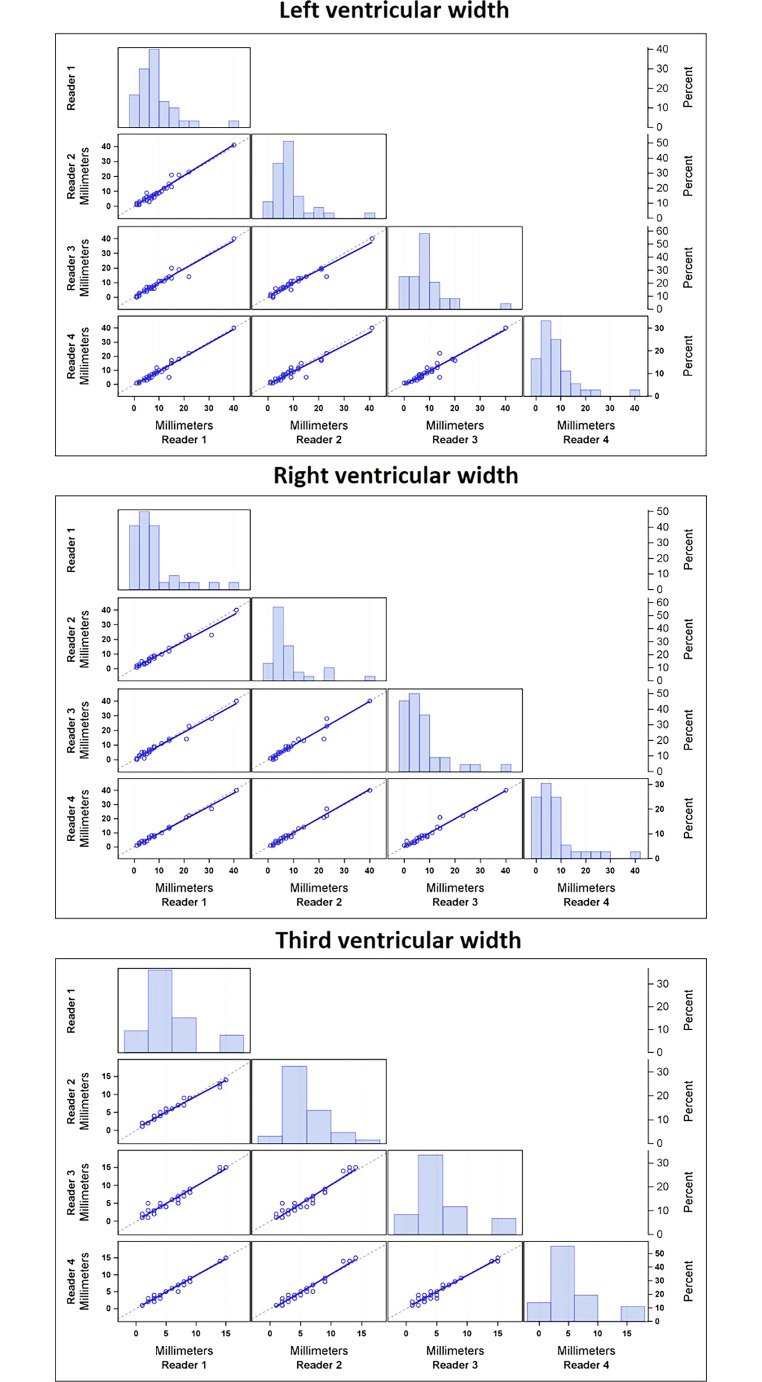
Scatter matrix for continuous variables showing the results for interrater reliability

Thereby, ultra-fast sequences demonstrated almost perfect agreement levels for interrater reliability assessment of diagnostic confidence (Gwet’s AC2 ≥ 0.886) and image quality (Gwet’s AC2 ≥ 0.942). For the assessment of ventricular width, excellent agreement (intraclass correlation coefficients ≥ 0.966) was observed at all defined measurement points (Table 4). The scatter matrices for the assessment of the ventricular width of both the lateral and the third ventricles are presented in Fig. 5. No significant differences were demonstrated in the correlation between age and image quality (S1). In the subgroup analysis of image quality, depending on the necessity for repetitive scans, no significant difference in image quality was observed between patients of different ages. However, patients who required repetitive scans tended to be younger, and when repetitions were necessary, mean image quality was rated 3.0 on the Likert-scale. In contrast, it was rated 4.0 when no repetitions were required. Further details are displayed in Table 5.

**Table 4:** Interrater reliability

Artifacts	Gwet’s AC_2_	0.967
Diagnostic confidence	Gwet’s AC_2_	0.886
Image quality	Gwet’s AC_2_	0.942
Left ventricle	ICC	0.966
Right ventricle	ICC	0.986
Third ventricle	ICC	0.979

ICC definitions according to Shroud & Fleiss

**Table 5:** Subgroup analysis of image quality, depending on necessary repetitions

		No repetition necessary		Repetition necessary		
Parameter	Reader	*n*	Descr. stat	*n*	Descr. stat	*p*-val.
Age	Overall	23	1182.6 (761.4)	13	872.6 (575.2)	0.178
Image quality	Reader 1	23	4.0 (2.0)	13	3.0 (0.0)	0.053
	Reader 2	23	4.0 (1.0)	13	3.0 (1.0)	0.122
	Reader 3	23	4.0 (2.0)	13	3.0 (1.0)	0.094
	Reader 4	23	4.0 (1.0)	13	3.0 (0.0)	0.111

Age displayed as mean in days (SD) and t‑test; Image quality displayed as median (IQR) and U‑Test

## Discussion

Here, we present a novel ultra-fast MRI protocol for assessing acute neuropediatric emergencies without anesthesia within seconds. In our cohort, we could demonstrate the feasibility of this protocol in clinical routine and that Deep-Learning-accelerated brain MRI yields most often good to excellent diagnostic confidence while reducing image acquisition time to a maximum of 47 s. Readers felt confident in using Deep-Learning-accelerated brain MRI to detect intracranial masses, evaluate the midline, determine the width of the ventricular system, and exclude acute intracranial pathology. There is an undersupply of pediatric MRIs in clinical routine due to the frequent need for anesthesia and the challenging conditions required. This means an experienced anesthetist, appropriate equipment, and extra time for preparation and follow-up are needed. Here, we demonstrate that using ultra-fast sequences has great potential to advance brain MRI in infants and young children without anesthesia.

As the ultra-fast protocol comprises three separate, independently applicable sequences, single sequences can be integrated into an already established clinical workflow. Due to highly increased acceleration factors, increasing agitation and restlessness may lead to excessive parallel imaging artefacts [16]. This has been recognized in 13 out of 36 cases. However, as sequences are very fast and can be easily repeated, in our experience, the MRI examination almost always results in acceptable image quality.

Thus, we could demonstrate that deep-learning-based sub-minute MRI examinations for pediatric emergency examinations seem possible, even with higher acceleration rates than already proven for the adult population, enabling ad hoc assessment of acute neuropediatric emergencies [17, 18]. Nevertheless, two examinations resulted in poor diagnostic confidence and two examination could not be performed due to agitation, resulting in the need for further follow-up. Therefore, especially in these cases, real-time MRI might be superior, and the additive use of both techniques, depending on the child’s restlessness, might achieve the best results. However, hardware and software are quite expensive, and not all techniques are simultaneously accessible at every institution.

As we demonstrated good to excellent diagnostic confidence in 73.6% of cases, this deep-learning-accelerated protocol may serve as a robust first-line MR imaging technique, replacing CT, to guide diagnostic decisions in emergency settings. Additionally, patients with congenital or post-inflammatory hydrocephalus who have undergone ventricular shunting can be adequately imaged with this technique.

The remarkably fast acquisition time will very likely decrease emotional stress in pediatric patients and their accompanying parents. Additionally, it will reduce treatment costs and hospitalization, as well as periprocedural risks accompanied with intravenous cannulation and general anesthesia [19–22]. Furthermore, it will lower the need for CT and thereby circumvent potential long-term risks associated with radiation exposure [23, 24]. The application of this sequence at 1.5 T is of particular clinical relevance as it extends this technology to nearly the entire patient population.

There are limitations: this is a single-center, retrospective study with a mixed patient sample and limited pathologies. Patients were examined with their parents seated to the side, their hands on the child’s body. Only in selected cases, when the child was agitated, and provided that safety clearance was obtained and informed consent was given, a parent was positioned inside the MRI tunnel, gently holding their child’s head [1]. Although this technique requires more time for preparation, it has proven to yield fewer motion artifacts and better overall image quality. Furthermore, as this was a feasibility study, the analysis mainly focused on subjective image quality evaluation, and intra-individual comparisons or assessments of interchangeability across different pathologies are missing. Besides, as our ultra-fast protocol consisted only of T2-weighted sequences in all three planes with a 5 mm slice thickness, the detection of small ischemic lesions or micro-hemorrhage was hardly possible and thus would require an adapted protocol. The underlying concept was to perform a rapid MRI of the neurocranium. While this method does not by any means provide a complete neuroradiologic workup, it is a useful ad hoc tool in emergency examinations as an alternative to CT scans, enabling the exclusion or assessment of the most relevant acute neuropediatric and especially traumatic complications. Therefore, implementing other ultra-fast imaging modalities, such as fluid-attenuated inversion recovery (FLAIR), diffusion-weighted imaging (DWI), and T1-weighted imaging, is crucial. Furthermore, diagnostic image quality was not rated with respect to a complete diagnostic neuroradiologic study. This would yield much lower image quality and diagnostic confidence scores due to higher slice thickness, possible blurring, and artifacts resulting from acceleration and patient motion. Thus, current sequences are most likely comparable only to clinical information, typically obtained from CT, without the child undergoing radiation exposure. Pathologies, like epileptic foci and subtle signs of diffuse axonal injury, could not be ruled out with this examination and would require further workup.

Besides, more extensive prospective studies on reproducibility across different disease types, as well as larger cohorts, are needed. As a next step, these ultra-fast sequences should be combined with a motion-correction algorithm to further improve image quality, as this will prevent necessary rescanning and improve diagnostic confidence.

## Conclusion

Employing Deep-Learning reconstruction algorithms in pediatric brain MRI will further enhance the scope of diagnostic imaging, increase patient comfort, reduce periprocedural risks and additionally lower healthcare costs.

## Supplementary Information

ESM1: Supplementary material 1

## Data Availability

No datasets were generated or analysed during the current study.
