# Supplementary material for: Ultra-Fast Sub-Minute Brain MRI with Deep-Learning-reconstruction for Anesthesia-Free Emergency Imaging in Children
Source: Clin Neuroradiol. 2026 Feb 19;36(2):681–90. doi: 10.1007/s00062-026-01627-7 (PMC13319877; doi:10.1007/s00062-026-01627-7)
Supplement: Supplementary file 1 — ESM1: Supplementary material 1 [file 62_2026_1627_MOESM1_ESM.docx]

| **Table S1** Correlation of age and image quality | | | |
| --- | --- | --- | --- |
| **Reader** | **Spearman's r** | **p-value** |  |
|  | | |  |
| Reader 1 | 0.251 | 0.139 |  |
|  | | |  |
| Reader 2 | 0.274 | 0.106 |  |
|  | | |  |
| Reader 3 | 0.183 | 0.286 |  |
|  | | |  |
| Reader 4 | 0.257 | 0.131 |  |
|  | | |  |
